# Immunogenicity and Safety of the Adjuvanted Recombinant Zoster Vaccine in Chronically Immunosuppressed Adults Following Renal Transplant: A Phase 3, Randomized Clinical Trial

**DOI:** 10.1093/cid/ciz177

**Published:** 2019-03-07

**Authors:** Peter Vink, Josep Maria Ramon Torrell, Ana Sanchez Fructuoso, Sung-Joo Kim, Sang-il Kim, Jeff Zaltzman, Fernanda Ortiz, Josep Maria Campistol Plana, Ana Maria Fernandez Rodriguez, Henar Rebollo Rodrigo, Magda Campins Marti, Rafael Perez, Francisco Manuel González Roncero, Deepali Kumar, Yang-Jen Chiang, Karen Doucette, Lissa Pipeleers, Maria Luisa Agüera Morales, Maria Luisa Rodriguez-Ferrero, Antonio Secchi, Shelly A McNeil, Laura Campora, Emmanuel Di Paolo, Mohamed El Idrissi, Marta López-Fauqued, Bruno Salaun, Thomas C Heineman, Lidia Oostvogels, Z-041 Study Group

**Affiliations:** 1 GlaxoSmithKline (GSK), Rockville, Maryland; 2 Bellvitge University Hospital, Barcelona; 3 Hospital Clínico San Carlos, Madrid, Spain; 4 Sungkyunkwan University, Canada; 5 Seoul St Mary’s Hospital, College of Medicine, Catholic University of Korea, Republic of Korea; 6 St Michael’s Hospital, University of Toronto, Ontario, Canada; 7 Helsinki University Hospital, Finland; 8 Hospital Clinic, University of Barcelona, Institut d’Investigacions Biomèdiques August Pi i Sunyer; 9 Hospital Ramón y Cajal, Madrid; 10 University Hospital Valdecilla, Santander; 11 Hospital Universitario Vall d’Hebron, Barcelona, Spain; 12 Social Security of Panama, Panama City; 13 Hospital Universitario Virgen Rocio, Sevilla, Spain; 14 University Health Network, Toronto, Ontario, Canada; 15 Chang Gung Memorial Hospital, Taoyuan, Taiwan; 16 University of Alberta, Edmonton, Canada; 17 UZ Brussel, Belgium; 18 Hospital Universitario Reina Sofia, Córdoba; 19 Hospital General Universitario Gregorio Marañón, Madrid, Spain; 20 Vita Salute San Reffaele University, Milan, Italy; 21 Canadian Center for Vaccinology, Izaak Walton Killam Health Centre and Nova Scotia Health Authority, Dalhousie University, Halifax, Canada; 22 GSK, Wavre, Belgium; 23 GSK, Rixensart, Belgium; 24 GSK, King of Prussia, Pennsylvania

**Keywords:** renal transplant, immunosuppression, herpes zoster vaccine, immunogenicity, safety

## Abstract

**Background:**

The incidence of herpes zoster is up to 9 times higher in immunosuppressed solid organ transplant recipients than in the general population. We investigated the immunogenicity and safety of an adjuvanted recombinant zoster vaccine (RZV) in renal transplant (RT) recipients ≥18 years of age receiving daily immunosuppressive therapy.

**Methods:**

In this phase 3, randomized (1:1), observer-blind, multicenter trial, RT recipients were enrolled and received 2 doses of RZV or placebo 1–2 months (M) apart 4–18M posttransplant. Anti–glycoprotein E (gE) antibody concentrations, gE-specific CD4 T-cell frequencies, and vaccine response rates were assessed at 1M post–dose 1, and 1M and 12M post–dose 2. Solicited and unsolicited adverse events (AEs) were recorded for 7 and 30 days after each dose, respectively. Solicited general symptoms and unsolicited AEs were also collected 7 days before first vaccination. Serious AEs (including biopsy-proven allograft rejections) and potential immune-mediated diseases (pIMDs) were recorded up to 12M post–dose 2.

**Results:**

Two hundred sixty-four participants (RZV: 132; placebo: 132) were enrolled between March 2014 and April 2017. gE-specific humoral and cell-mediated immune responses were higher in RZV than placebo recipients across postvaccination time points and persisted above prevaccination baseline 12M post–dose 2. Local AEs were reported more frequently by RZV than placebo recipients. Overall occurrences of renal function changes, rejections, unsolicited AEs, serious AEs, and pIMDs were similar between groups.

**Conclusions:**

RZV was immunogenic in chronically immunosuppressed RT recipients. Immunogenicity persisted through 12M postvaccination. No safety concerns arose.

**Clinical Trials Registration:**

NCT02058589.

## Teaser

The adjuvanted recombinant zoster vaccine was immunogenic in renal transplant recipients receiving daily immunosuppressive treatment. Humoral and cell-mediated immune responses persisted through 1 year postvaccination. Vaccination had no impact on renal function or rejection rate. No safety concerns arose.

**(See the Editorial Commentary by Miller on pages 191–2.)**

Herpes zoster (HZ) results from reactivation of latent varicella zoster virus (VZV), and usually presents as a painful dermatomal rash [1]. The most common complication of HZ is postherpetic neuralgia, chronic pain that can persist for months or even years after resolution of the zoster rash [2].

Diminished cellular immunity to VZV increases the risk of HZ [3]. Compared to healthy individuals, persons with impaired cellular immunity, especially those receiving immunosuppressive therapies after organ transplantation, are at increased risk of developing HZ [4–10]. In particular, solid organ transplant (SOT) patients have a mixed, but mainly cellular immune deficit, induced by chronic immunosuppressive therapy required to prevent organ rejection.

The observed HZ incidence rates in SOT recipients [4–6] are up to 9 times higher compared with those in the general population (22–28 vs 3–5/1000 person-years, respectively) [7, 8]. However, HZ incidence varies depending on the type of transplanted organ [6].

Although efficacy of vaccines in SOT recipients is often suboptimal, immunization against common infectious diseases is currently recommended for SOT candidates and recipients to reduce the risk of infections [9]. While infection may stimulate the immune system, leading to an increased risk of rejection [11], there is very limited evidence of a correlation between vaccination and allograft rejection. Nevertheless, this remains a general concern for transplant physicians [9, 12].

Currently, 2 vaccines are licensed for the prevention of HZ in different regions worldwide. The live attenuated zoster vaccine (Zostavax) is, however, contraindicated in immunosuppressed individuals, including SOT recipients [13].

The adjuvanted recombinant zoster vaccine (RZV; Shingrix) is a nonlive vaccine that consists of a truncated form of VZV glycoprotein E (gE) and the GSK AS01_B_ Adjuvant System. RZV is licensed for the prevention of HZ and postherpetic neuralgia in adults ≥50 years of age [14, 15]; it is highly immunogenic and demonstrated >90% efficacy against HZ in all age groups among adults aged ≥50 years, 68% efficacy in autologous hematopoietic stem cell transplant (HSCT) recipients ≥18 years of age, and 87% efficacy in a post hoc analysis in patients ≥18 years of age with hematologic malignancies. The safety profile of RZV was clinically acceptable in these populations [14–20].

In this study, we evaluated the immunogenicity and safety of RZV in renal transplant (RT) patients ≥18 years of age receiving daily immunosuppressive therapy. As the transplant community has a long-established renal allograft monitoring program and because the immunosuppressive therapies used for RTs are also used for other SOTs, RT was selected as a model for SOTs.

## METHODS

### Study Design and Participants

This was a phase 3, randomized, observer-blind, placebo-controlled, multicenter trial, conducted in 9 countries (Belgium, Canada, Czech Republic, Finland, Italy, Panama, Republic of Korea, Spain, and Taiwan) between March 2014 and April 2017. RT recipients were randomized 1:1 to receive 2 doses of RZV or placebo 1–2 months (M) apart, at visits defined as M0 and M1 visits. The randomization algorithm included the following minimization factors: age (18–29, 30–49, or ≥50 years), gender, participant’s highest panel reactive antibody (PRA) or calculated PRA (cPRA) score at/prior to transplant, and maintenance immunosuppressive therapy (use of mycophenolate compounds, calcineurin inhibitors or sirolimus, or corticosteroids). RT patients ≥18 years of age were eligible for participation at 4–18M posttransplantation if they had received an ABO-compatible allograft, had stable renal function, and were free of any allograft rejection in the 3M preceding the first vaccination.

RT recipients were excluded from participation if they had a primary kidney disease (PKD) known to have a high incidence of recurrence, a previous allograft loss due to recurrent PKD, multiple organs transplanted, or a condition that could interfere with study-required evaluations. Persons were also excluded if they had any systemic autoimmune or potential immune-mediated disease (pIMD) listed in Supplementary Table 1 (exceptions are listed in the Supplementary Text 1B), had clinical history of HZ or varicella, or received HZ/varicella vaccination within the 12M preceding the first dose of study vaccine/placebo. The full list of inclusion and exclusion criteria is provided in Supplementary Text 1.

All participants provided written informed consent at enrollment. The study protocol was reviewed and approved by independent ethics committees or institutional review boards. The study was conducted in accordance with the Declaration of Helsinki and the principles of Good Clinical Practice. The study is registered at ClinicalTrials.gov (NCT02058589). Anonymized individual participant data and study documents can be requested for further research at www.clinicalstudydatarequest.com.

### Study Vaccine

Study participants received 2 intramuscular doses of RZV or placebo 1–2M apart in a deltoid muscle. Each 0.5-mL dose of RZV contained 50 μg of recombinant VZV gE antigen and the GSK proprietary AS01_B_ Adjuvant System (containing 50 μg of 3-O-desacyl-4’-monophosphoryl lipid A, 50 μg of *Quillaja saponaria* Molina, fraction 21 [QS21, licensed by GSK from Antigenics LLC, a wholly owned subsidiary of Agenus Inc, a Delaware, USA corporation], and liposome). Each 0.5-mL dose of placebo contained 20 mg lyophilized sucrose reconstituted with 150 mM sodium chloride solution.

### Assessment of Immunogenicity

Humoral immunogenicity was assessed from blood samples collected from each participant at prevaccination (M0 visit), 1–2M post–dose 1 (M1 visit), 1M post–dose 2 (M2 visit), 6M post–dose 2 (M7 visit), and 12M post–dose 2 (M13). Anti-gE antibody concentrations were measured by anti-gE enzyme-linked immunosorbent assay with a technical cutoff of assay quantification of 97 mIU/mL. Cell-mediated immunogenicity (CMI) was evaluated in a subset of participants at the M0, M2, and M13 visits. The frequencies of gE-specific CD4[2+] T cells (CD4^ +^ T-cells expressing at least 2 activation markers of the 4 markers assessed: interferon-γ, interleukin 2, tumor necrosis factor–α, and CD40 ligand) were measured, after in vitro stimulation with a pool of peptides covering the gE ectodomain, by intracellular cytokine staining and detection by flow cytometry as described previously [20]. The cutoff for the CMI vaccine response analysis was 320 positive cells per 10^6^ CD4^ +^ T cells counted.

### Assessment of Reactogenicity and Safety

Diary cards were provided to all participants to record solicited local (pain, redness, and swelling at the injection site) and general (fever [body temperature ≥37.5°C/99.5°F], headache, fatigue, gastrointestinal symptoms [nausea, vomiting, diarrhea, and/or abdominal pain], myalgia, and shivering) adverse events (AEs) during 7 days (D) after each vaccination, and unsolicited AEs during 30D after each vaccination. Solicited general AEs, as well as unsolicited AEs, were also recorded during 7D before first vaccination to evaluate the baseline values resulting from the underlying condition of participants. AEs were graded from 0 (none/normal) to 3 (severe). Grade 3 AEs were defined as preventing normal activity (for all unsolicited AEs, and for headache, fatigue, gastrointestinal symptoms, myalgia, and shivering), as significant pain at rest, and preventing normal everyday activities (for pain) and having a surface diameter >100 mm (for injection-site redness and swelling).

All solicited local AEs were considered causally related to vaccination. The causal relationship to vaccination of all other AEs occurring postvaccination was assessed by the investigator. Allograft function (by routine serum creatinine measurements) was reported from first vaccination to study end.

Serious AEs (SAEs), including biopsy-proven allograft rejections, and pIMDs were recorded from first vaccination to M13. In addition, SAEs related to study participation were recorded from enrollment to study end. If a clinical event was suspicious for HZ per the investigator’s judgement, it was considered a suspected case of HZ. Suspected cases and HZ complications were recorded from first vaccination to study end and constituted AEs/SAEs, as appropriate.

### Outcomes

Study objectives and their evaluation criteria are presented in Table 1.

**Table 1.** Study Objectives

No.	Objective	Success Criterion
Primary objectives		
I	To evaluate VRR for the anti-gE humoral immune response at M2, following 2 doses of RZV in all participants.	The objective was met if the LL of the 95% CI of the VRR^a^ for anti-gE antibody concentrations at M2 in the RZV group was ≥60%.
II	To evaluate the safety of RZV, as compared to placebo, from first vaccination until 30 days after last vaccination in all participants.	Descriptive
Secondary objectives		
III	To evaluate the anti-gE humoral immune response at M2, following 2 doses of RZV, as compared to placebo, in all participants.	The objective was met if the LL of the 95% CI of the geometric mean ratio (RZV over placebo) of anti-gE concentrations at M2 was >3.
IV	To characterize anti-gE humoral immune responses at M0, M1, M2, M7, and M13, within the RZV and placebo groups, in all participants.	Descriptive
V	To evaluate VRR for gE-specific CD4^ +^ T-cell–mediated immune responses at M2, following a 2-dose administration of the RZV, in the CMI subcohort.	The objective was met if the LL of the 95% CI of the VRR^b^ for gE-specific CD4[2+] T-cell frequencies at M2 in the RZV group was ≥50%.
VI	To evaluate gE-specific CD4^ +^ T-cell–mediated immune response at M2 following 2 doses of RZV, as compared to placebo, in the CMI subcohort.	The objective was met if the LL of the 95% CI of the geometric mean ratio (RZV over placebo) of gE-specific CD4[2+] T-cell frequencies at M2 was >1.
VII	To characterize gE-specific CD4^ +^ T-cell–mediated immune responses at M0, M2, and M13, within the RZV and placebo groups, in the CMI subcohort.	Descriptive
VIII	To evaluate the safety of the vaccine, as compared to placebo, from 30 days after last vaccination until study end in all participants.	Descriptive

Abbreviations: CD4[2+], CD4^ +^ T cells expressing at least 2 activation markers of the 4 markers assessed (interferon-γ, interleukin 2, tumor necrosis factor–α, and CD40 ligand); CI, confidence interval; CMI, cell-mediated immunogenicity; gE, glycoprotein E; LL, lower limit; M, study month; placebo, participants receiving placebo; RZV, participants receiving the recombinant adjuvanted herpes zoster vaccine; VRR, vaccine response rate.

^a^VRR in terms of anti-gE humoral response was defined as the percentage of participants with postvaccination anti-gE concentrations (*i*) ≥4-fold the technical cutoff of assay quantification (for initially seronegative participants) or (*ii*) ≥4-fold the prevaccination concentration (for initially seropositive participants).

^b^VRR in terms of CD4[2+] T-cell response was defined as the percentage of participants with postvaccination CD4[2+] T-cell frequencies ≥2-fold the cutoff (320 positive cells per 10^6^ CD4^ +^ T cells counted) (for participants initially below the cutoff) or ≥2-fold the prevaccination CD4[2+] T-cell frequencies (for participants initially above the cutoff).

### Statistical Analyses

Safety and reactogenicity were assessed in the total vaccinated cohort (TVC), which included all participants with at least 1 administered/documented vaccine dose. The analysis of humoral immunogenicity during the vaccination (up to M2) and persistence (up to M13) phases were performed on the applicable according-to-protocol cohorts, which included all participants who complied with the protocol-specified procedures and for whom data were available. The analysis of gE-specific CMI during the vaccination and persistence phases was performed on the applicable according-to-protocol cohorts of the CMI subcohort, which included the first enrolled participants from designated sites that had access to a GSK-validated peripheral blood mononuclear cell processing facility. Further details on statistical methods, including sample size calculation, are provided in Supplementary Text 2.

## RESULTS

### Study Participants

**Figure 1.**
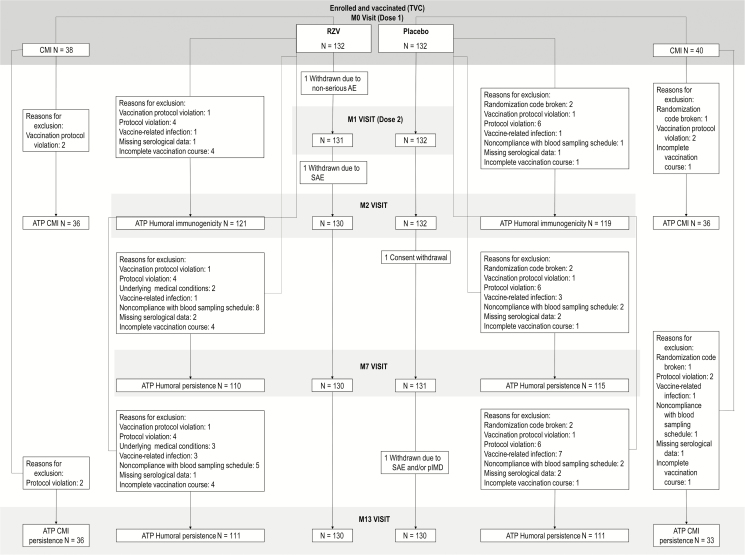
Participant flowchart. Abbreviations: AE, adverse event; ATP, according to protocol; CMI, cell-mediated immunogenicity; M, study month; n, number of participants in each category; pIMD, potential immune-mediated disease; Placebo, participants receiving placebo; RZV, participants receiving the recombinant adjuvanted herpes zoster vaccine; SAE, serious adverse event; TVC, total vaccinated cohort.

A total of 264 RT patients (RZV: 132, placebo: 132) were included in the TVC. Of these, 260 (RZV: 130, placebo: 130) participated through to the last study visit at M13 (Figure 1). Demographic characteristics were balanced between study groups (Table 2). At dose 1, the mean ages were 52.3 and 52.4 years in the RZV and placebo groups, respectively. Most participants were male (RZV: 71.2%, placebo: 68.9%) and white (RZV: 66.7%, placebo: 73.5%).

**Table 2.** Summary of Demographic Characteristics (Total Vaccinated Cohort)

Characteristic	Parameter or Category	RZV (N = 132)	Placebo (N = 132)
Age at dose 1, y	Mean ± SD	52.3 ± 12.5	52.4 ± 12.8
Gender	Female	38 (28.8)	41 (31.1)
	Male	94 (71.2)	91 (68.9)
Age group	18–49 y	48 (36.4)	49 (37.1)
	≥50 y	84 (63.6)	83 (62.9)
Geographic ancestry	White–Caucasian/European heritage	88 (66.7)	97 (73.5)
	Asian–East Asian heritage	20 (15.2)	22 (16.7)
	Asian–South East Asian heritage	10 (7.6)	3 (2.3)
	African heritage/African American	3 (2.3)	1 (0.8)
	White–Arabic/North African heritage	2 (1.5)	2 (1.5)
	Asian–Central/South Asian heritage	1 (0.8)	2 (1.5)
	Asian–Japanese heritage	0 (0.0)	1 (0.8)
	Other	8 (6.1)	4 (3.0)
PRA/cPRA score	0%–19%	117 (88.6)	117 (88.6)
	20%–79%	13 (9.8)	12 (9.1)
	80%–100%	2 (1.5)	3 (2.3)
Immunosuppressive therapy	CIS + CS + MC	100 (75.8)	102 (77.3)
	CIS + MC	23 (17.4)	22 (16.7)
	CIS + CS	7 (5.3)	8 (6.1)
	Other combinations^a^	2 (1.5)	0 (0.0)

Data are presented as no. (%) except for the ‘Age at dose 1’ data.

Abbreviations: CIS, calcineurin inhibitor or sirolimus; cPRA, calculated panel reactive antibody; CS, corticosteroids; MC, mycophenolate compound; N, number of vaccinated participants; placebo, participants receiving placebo; PRA, panel reactive antibody; RZV, participants receiving the recombinant adjuvanted herpes zoster vaccine; SD, standard deviation.

^a^Other immunosuppressive combinations are not included in the table secondary to small sample size.

### Immunogenicity

#### Humoral Immunogenicity

**Figure 2.**
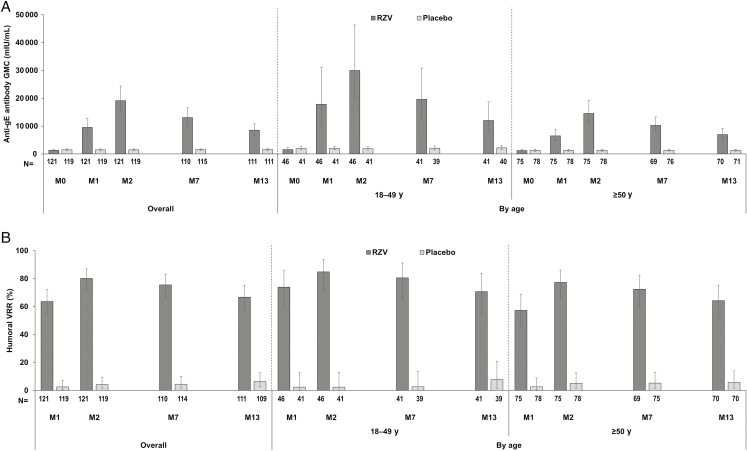
Humoral immune responses (according-to-protocol cohort for humoral immunogenicity). *A*, Anti-gE antibody geometric mean concentration; *B*, humoral vaccine response rate. Vaccine response rate in terms of anti–glycoprotein E (gE) humoral response was defined as the percentage of participants with postvaccination anti-gE concentrations (*i*) ≥4-fold the technical cutoff of assay quantification (for initially seronegative participants) or (*ii*) ≥4-fold the prevaccination concentration (for initially seropositive participants). Abbreviations: GMC, geometric mean concentration; M, study month; N, number of participants in the according-to-protocol cohort for humoral immunogenicity; Placebo, participants receiving placebo; RZV, participants receiving the recombinant adjuvanted herpes zoster vaccine; VRR, vaccine response rate.

Both confirmatory objectives on humoral immunogenicity were met: The humoral vaccine response rate (VRR) in the RZV group was 80.2% (95% confidence interval [CI], 71.9%–86.9%) at M2 (success criterion: lower limit [LL] of 95% CI ≥60%), and the adjusted anti-gE antibody geometric mean concentration (GMC) ratio (RZV over placebo) was 14.00 (95% CI, 10.90–17.99; *P* < .0001) at M2 (success criterion: LL of 95% CI >3) (Figure 2).

Among RZV recipients, anti-gE antibody GMCs increased from 1354.4 mIU/mL (95% CI, 1118.3–1640.4 mIU/mL) at prevaccination to 19 163.8 mIU/mL (95% CI, 15 041.5–24 416.0 mIU/mL) at M2 and persisted through M13 at 8545.1 mIU/mL (95% CI, 6753.7–10 811.5 mIU/mL). Postvaccination anti-gE antibody GMCs and humoral VRRs in the placebo group remained near the prevaccination level. Across time points, no apparent differences in anti-gE antibody GMCs were seen linked to the types of maintenance immunosuppressive therapy (Supplementary Table 2). At each time point, humoral immune responses appeared higher in the 18–49 years of age cohort than in the ≥50 years of age cohort (Figure 2). Within the 18–49 years of age cohort, post hoc analyses revealed humoral immune responses similar for ages 18–29 and 30–49 years (Supplementary Table 3).

#### Cell-mediated Immunogenicity

**Figure 3.**
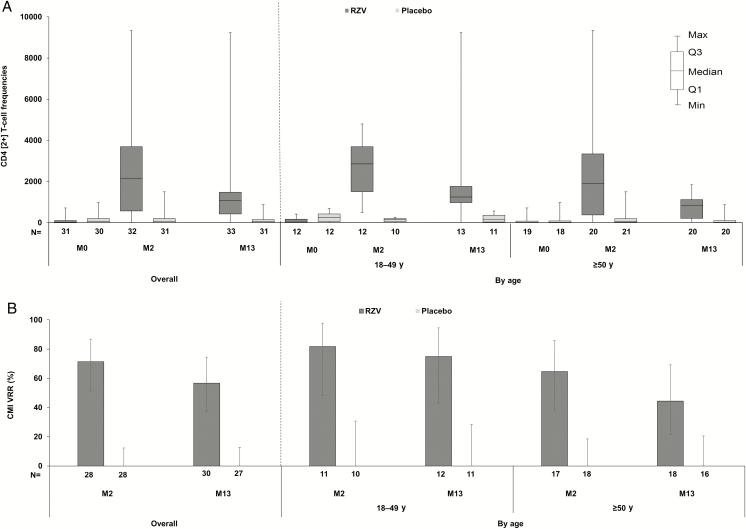
Cell-mediated immune responses (according-to-protocol cohort for cell-mediated immunogenicity). *A*, CD4[2+] T-cell frequencies; *B*, cell-mediated vaccine response rate. Vaccine response rate in terms of CD4[2+] T-cell response was defined as the percentage of participants with postvaccination CD4[2+] T-cell frequencies (*i*) ≥2-fold the cutoff (320 positive cells per 10^6^ CD4^ +^ T cells counted) (for participants initially below the cutoff) or (*ii*) ≥2-fold the prevaccination CD4[2+] T-cell frequencies (for participants initially above the cutoff). Abbreviations: CD4[2+], CD4^ +^ T cells expressing at least 2 activation markers of the 4 markers assessed (interferon-γ, interleukin 2, tumor necrosis factor–α, and CD40 ligand); CMI, cell-mediated immunogenicity; M, study month; N, number of participants in the according-to-protocol cohort for cell-mediated immunogenicity; Placebo, participants receiving placebo; RZV, participants receiving the recombinant adjuvanted herpes zoster vaccine; VRR, vaccine response rate.

Though CMI was measured in a limited subset of participants, both confirmatory CMI objectives were met: The VRR for CMI responses was 71.4% (95% CI, 51.3%–86.8%) in the RZV group at M2 (success criterion: LL of 95% CI ≥50%) (Figure 3); the geometric mean ratio (RZV over placebo) of gE-specific CD4[2+] T-cell frequencies was 17.26 (95% CI, 5.92–50.36; *P* < .0001) at M2 (success criterion: LL of 95% CI >1).

At prevaccination, median CD4[2+] T-cell frequencies were 21.2 and 59.7 in the RZV and placebo groups, respectively. In the RZV group, median frequencies increased to 2149.0 at M2 and remained significantly elevated over prevaccination baseline at M13. Postvaccination CD4[2+] T-cell frequencies and CMI VRRs in the placebo group remained near the prevaccination level. At each time point, CMI responses appeared to be higher in the 18–49 years of age cohort (Figure 3). Within the 18–49 years of age cohort, post hoc analyses revealed CMI responses similar for the age 18–29 and 30–49 years of age groups (Supplementary Table 4).

### Reactogenicity and Safety

**Figure 4.**
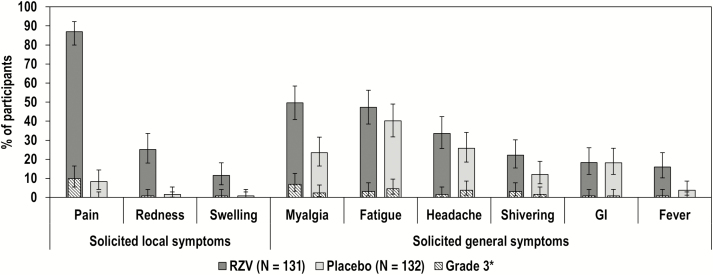
Reactogenicity in the total vaccinated cohort. Error bars indicate 95% confidence intervals. Fever was defined as body temperature ≥37.5°C. Abbreviations: GI, gastrointestinal symptoms (nausea, vomiting, diarrhea, and/or abdominal pain); N, number of participants with at least 1 documented vaccine administration; Placebo, participants receiving placebo; RZV, participants receiving the recombinant adjuvanted herpes zoster vaccine. *Fever was not graded in this study. For fever, body temperatures >39°C are presented as grade 3.

During the 7D postvaccination period, injection site pain was the most frequent solicited local symptom, reported by 114 (87.0%) of RZV and 11 (8.3%) of placebo participants (Figure 4). Solicited local symptoms in RZV recipients had a median duration of 4D or less.

During the 7D postvaccination period, myalgia and fever were reported at higher rates by RZV compared to placebo participants. Myalgia was reported by 65 (49.6%) RZV participants and by 31 (23.5%) placebo participants; and fever by 21 (16.0%) RZV participants and 5 (3.8%) placebo participants (Figure 4). Myalgia, shivering, and fever appeared to be more frequently reported post- than prevaccination in the RZV group (Supplementary Figure 1*B* and 1*C*).

During the 7D prevaccination period, at least 1 unsolicited symptom was reported by 9 (6.8%) RZV participants and 7 (5.3%) placebo participants. No grade 3 unsolicited AEs were reported in either study group during this time period.

During the longer, 30D postvaccination period, at least 1 unsolicited symptom (any grade) was reported by 51 (38.6%) RZV participants and 44 (33.3%) placebo participants. Grade 3 unsolicited symptoms were reported by 7 (5.3%) RZV and 5 (3.8%) placebo participants (Table 3).

**Table 3.** Safety and Reactogenicity (Total Vaccinated Cohort)

		RZV (N = 132^a^)		Placebo (N = 132)	
AEs		No.	% (95% CI)	No.	% (95% CI)
Reported during the 7D prevaccination period					
Unsolicited AEs	Any grade	9	6.8 (3.2–12.5)	7	5.3 (2.2–10.6)
	Grade 3	0	—	0	—
Reported during the 7D postvaccination period					
Solicited local AEs	Any grade	115	87.8 (80.9–92.9)	12	9.1 (4.8–15.3)
	Grade 3	14	10.7 (6.0–17.3)	0	0 (0–2.8)
Solicited general AEs	Any grade	90	68.7 (60.0–76.5)	73	55.3 (46.4–64.0)
	Grade 3	13	9.9 (5.4–16.4)	11	8.3 (4.2–14.4)
Reported during the 30D postvaccination period					
Unsolicited AEs	Any grade	51	38.6 (30.3–47.5)	44	33.3 (25.4–42.1)
	Grade 3	7	5.3 (2.2–10.6)	5	3.8 (1.2–8.6)
	Related^b^–any grade	7	5.3 (2.2–10.6)	3	2.3 (0.5–6.5)
	With medically attended visits	34	25.8 (18.5–34.1)	29	22.0 (15.2–30.0)
Reported from first vaccination up to 30D after last vaccination					
SAEs	All	6	4.5 (1.7–9.6)	5	3.8 (1.2–8.6)
	Biopsy-proven allograft rejection	0	0	0	0
pIMDs	All	0	—	0	—
Reported from 30D after last vaccination up to study end					
SAEs	All	21	15.9 (10.1–23.3)	29	22.0 (15.2–30.0)
	Biopsy-proven allograft rejection	4	3.0	7	5.3
pIMDs	All	4	3.0 (0.8–7.6)	2	1.5 (0.2–5.4)
Reported from first vaccination up to study end					
SAEs	At least 1 symptom	26	19.7 (13.3–27.5)	33	25.0 (17.9–33.3)
	Related^b^	0	0 (0.0–2.8)	1	0.8 (0.0–4.1)
	Fatal	1	0.8	1	0.8
pIMDs	At least 1 symptom	4	3.0 (0.8–7.6)	2	1.5 (0.2–5.4)
Serum creatinine increase	>1.5-fold	4	3.1	4	3.0
	>1.75-fold	3	2.3	2	1.5
	>2-fold	2	1.5	1	0.8

Data are overall/participant. No. and % indicate the number and percentage of participants reporting at least 1 event; N indicates the number of participants with at least 1 documented (solicited AEs) or administered (other AEs) dose.

Abbreviations: AE, adverse event; CI, confidence interval; D, days; pIMD, potential immune-mediated disease; Placebo, participants receiving placebo; RZV, participants receiving the recombinant adjuvanted herpes zoster vaccine; SAE, serious adverse event.

^a^For the 7D postvaccination period and for creatinine fold increase (N = 131).

^b^Related indicates potentially causally related to vaccination per investigator assessment.

From first vaccination through M13, SAEs were reported by 26 (19.7%) RZV and 33 (25.0%) placebo participants. Of these, 3 SAEs (febrile neutropenia, mucosal inflammation, and Burkitt lymphoma) were considered as causally related to vaccination and were reported by 1 placebo recipient. Overall, the most frequent SAEs classified by Medical Dictionary for Regulatory Activities System Organ Class were “infections and infestations.”

Throughout the entire study, 1 fatality (0.8%) was reported in each of the study groups (RZV: purulent meningitis; placebo: coronary artery disease complicated by vein graft thrombosis and myocardial infarction; Table 3). Neither was considered causally related to vaccination by the investigator.

No biopsy-proven renal allograft rejections occurred from first vaccination up to 30D after the last dose in either group. Throughout the entire study, 4 (3.0%) and 7 (5.3%) biopsy-proven rejections occurred in the RZV and placebo groups, respectively (Table 3). Of these, 1 in the RZV group and all 7 in the placebo group occurred in participants with low rejection risk based on PRA/cPRA predictions (PRA/cPRA, 0–19%).

Serum creatinine level increases >1.5-fold were detected in 4 (3.1%) of RZV and 4 (3.0%) of placebo recipients. Percentages of participants with >1.75-fold or >2-fold increases were also similar in the 2 study groups (Table 3).

No pIMDs were reported from first vaccination up to 30D after the last dose in either group. Through M13, pIMDs were reported by 4 (3.0%) RZV and 2 (1.5%) placebo participants (Table 3).

In the TVC, 3 (2.3%) RZV recipients and 7 (5.3%) placebo participants reported suspected HZ episodes. One of the episodes occurred in a participant who had not yet received both RZV doses.

## DISCUSSION

**Figure 5.**
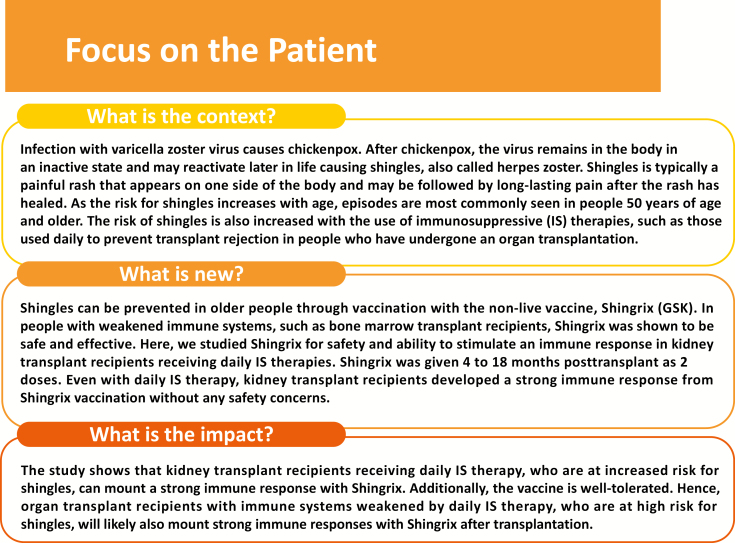
“Focus on the patient” section.

Our study demonstrates that RZV was immunogenic in RT patients aged ≥18 years under chronic daily immunosuppression. Humoral and cellular immune responses to vaccination persisted through 1 year after vaccination. No safety concerns were identified in this study. A results summary contextualizing the results and potential clinical relevance is provided in Figure 5 to assist communications to the patient.

RZV induced robust humoral and cell-mediated immune responses to gE when administered 4–18M after RT. Vaccination took place when RT maintenance immunosuppression levels were achieved, and prior to the rise of HZ incidence in this population. As seen in other RZV trials in immunocompromised populations such as autologous HSCT recipients [19], patients with solid tumors [21], and patients with hematologic malignancies [16], RZV was found to be immunogenic as shown by high VRR for both humoral and cellular immune responses, as well as by anti-gE antibody GMC ratios and CD4[2+] T-cell frequency ratios.

Anti-gE antibody GMCs and humoral VRRs at 1M and 12M post–dose 2 were in similar ranges with those in autologous HSCT recipients ≥18 years of age [19]. In this study, both RZV-induced humoral and cellular immune responses appeared to be higher in the younger (18–49 years of age) compared to the older (≥50 years of age) age groups across all time points. In the older age group of this study, humoral GMCs and VRRs, as well as CMI VRRs, at 1M and 12M post–dose 2 were lower than in immunocompetent adults of the same age [20]. However, gE-specific CD4[2+] T-cell frequencies at 1M and 12M post–dose 2 in the older age group were in similar ranges with those in immunocompetent adults ≥50 years of age [20]. As VZV-specific cellular immunity is believed to be the main mechanistic driver of protection against HZ [22], RZV vaccination is expected to reduce the risk of HZ in RT recipients [14, 15]. The responses of adults ≥50 years of age in our study are likely a result of the combined effects of immunosenescence and the use of maintenance immunosuppressive therapy.

Though the number of participants in each subgroup was low, our results indicate that RZV-induced humoral immune responses were similar in range across the different immunosuppressive regimens assessed.

In line with the reactogenicity profile of RZV in the pivotal phase 3 efficacy trials, RZV recipients reported solicited local symptoms more frequently than placebo recipients [14, 15]. Compared to placebo, the frequency of solicited general symptom reporting by RZV recipients increased only for myalgia after dose 1 and for myalgia, shivering, and fever after dose 2. Solicited AEs were primarily mild to moderate and transient in nature. In the RZV group, only 1 participant (<1%) withdrew from the study before receiving dose 2, due to an AE (fever) that persisted for 2 days after receiving RZV dose 1.

Overall, no apparent differences were observed between study groups in the occurrence of unsolicited AEs, SAEs (including fatalities), pIMDs, biopsy-proven allograft rejections, or allograft function changes. Overall, the reported events are consistent with the background disease or concomitant medications. Indeed, RZV does not impact allograft function, as observed through creatinine measurements or rejection rate. A lower rate of suspected HZ cases was reported in RZV vs placebo recipients (3 vs 7 suspected cases).

Taken together with the clinically acceptable safety profile, the benefit-risk profile of RZV in RT recipients appears favorable, though vaccine effectiveness in this population has not been established.

Our results should be interpreted considering the study’s strengths and limitations. Study strengths include the fact that randomization was performed using several minimization factors leading to comparable baseline characteristics between the 2 study groups. Considering the high rate of solicited general AEs in the RT population, these were also recorded for 7D before vaccination, to indicate the increase of such AEs due to vaccination. Renal allograft function and rejections were followed up for 1 year. As the study was carried out in a limited number of geographic regions, the racial heterogeneity was not very broad. However, in an earlier study, race did not appear to impact RZV immunogenicity [20]. Furthermore, the analysis by age and by immunosuppressive treatment regimen should be interpreted with caution as the number of participants in each of these subgroups was low. While this study was not designed to establish the immunologic correlates of protection or determine the vaccine efficacy in this population, the study’s safety profile and robust immune responses suggest a favorable benefit-risk assessment for RZV in RT recipients.

In conclusion, RZV was immunogenic in RT recipients receiving daily immunosuppressive therapy. Humoral and cellular immunogenicity persisted through the 1-year evaluation, while no vaccine-related concerns were identified. No apparent differences were observed between RZV and placebo recipients for allograft function and rejections.

## Supplementary Data

Supplementary materials are available at *Clinical Infectious Diseases* online. Consisting of data provided by the authors to benefit the reader, the posted materials are not copyedited and are the sole responsibility of the authors, so questions or comments should be addressed to the corresponding author.

ciz177_suppl_Supplementary_Materials

## Notes

***Acknowledgments.*** The authors thank all study participants; the Zoster-041 study group members and study nurses and coordinators involved in the Zoster-041 trial; the GlaxoSmithKline (GSK) study delivery manager (Lakshmi Kumar); the GSK Case Management Group Senior Clinical Safety Associate (Anne-Sophie Vilain); the GSK Biostatistics and Statistical Programming team (including Catherine Dettori and Gregory Catteau); and the GSK Clinical Immunology Platform (including Michel Janssens, Olivier Jauniaux and Valérie Berthold). Medical writing services were provided by Silviu Bercea and Alpár Pöllnitz (XPE Pharma & Science on behalf of GSK). Editorial assistance and publication coordination were provided by Divya Kesters (XPE Pharma & Science on behalf of GSK). Zostavax is a trademark of Merck Sharp & Dohme. Shingrix is a trademark of the GSK group of companies.

***Zoster-041 study group nonauthor collaborators.*** Caldara Rossana, Carmellini Mario, Chen Yen-Ta, Garibotto Giacomo, González Montes Esther, Kanaan Nada, Kuypers Dirk, Lin Cheng-Chia, Maggiore Umberto, Navratil Pavel, Van der Tol Arjan, Villate Navarro José Ignacio, Virgilio Bice, and Wu Ming-Ju.

***Author contributions.*** T. C. H., L. O., P. V., and M. E. I. were involved in the conception or design of the study. M. C. M., L. C., A. M. F. R., F. M. G. R., S.-J. K., S.-I. K., M. L.-F., S. A. M., F. O., R. P., J. M. R. T., H. R. R., A. S. F., J. Z., K. D., M. L. R.-F., M. L. A. M., E. D. P., B. S., J. M. C. P., D. K., Y.-J. C., L. P., and A. S. contributed to data collection or data generation. P. V., M. E. I., T. C. H., L. O., L. C., S. A. M., J. Z., M. L.-F., E. D. P., B. S., and J. M. C. P. contributed to data analysis or data interpretation. All authors reviewed the draft critically, approved the final version to be submitted, and take accountability for all aspects of the published work.

***Financial support*** . This work was funded by GlaxoSmithKline Biologicals SA. GSK Biologicals SA was involved in all stages of the conduct and analysis of the studies. GlaxoSmithKline Biologicals SA covered the costs associated with the development and the publishing of the present manuscript.

***Potential conflicts of interest.*** P. V., L. C., E. D. P., M. E. I., M. L.-F., B. S., T.C.H., and L. O. were employees of the GSK group of companies at the time this study was designed, initiated, and/or conducted. P. V., L. C., E. D. P., M. E. I., M. L.-F., and B. S. are currently employed by the GSK group of companies. L. O. is an employee of CureVacAG as of 1 March 2018 and is an inventor on a patent application related to the vaccine used in this study. T. C. H. was a paid consultant for the GSK group of companies during the development of this manuscript and is an inventor on a patent application related to the vaccine used in this study. L. O., T. C. H., P. V., B. S., E. D. P., L. C., and M. E. I. hold shares or stock options in the GSK groups of companies as part of their current or former employee remuneration. D. K. and K. D. declare that their institution received a clinical trial grant from GSK. D. K. has received speaker honoraria from Merck and personal fees for participation in GSK advisory boards. M. C. M. declares that her institution received grants for the conduct of clinical trials by GSK, Sanofi, and Novartis, and has received consulting fees from GSK, Pfizer, Sanofi, and Merck to participate in expert meetings and symposiums. S.-I. K. has received personal fees from Health Insurance Review and Assessment Service. S. A. M. received honoraria for participation in scientific advisory boards from GSK, Pfizer, Sanofi Pasteur, and Merck and for provision of accredited Continuing Medical Education to healthcare professionals on adult immunization and zoster vaccines. S. A. M.’s institution has a clinical trial contract with Novartis and has received research grants for conduct of clinical trials by GSK, Merck, Pfizer and Sanofi Pasteur. All other authors report no potential conflicts of interest. All authors have submitted the ICMJE Form for Disclosure of Potential Conflicts of Interest. Conflicts that the editors consider relevant to the content of the manuscript have been disclosed.

Presented in part: IDWeek, San Diego, California, 4–8 October 2017; IDWeek 2018, San Francisco, California, 3–7 October 2018.
