# Supplementary material for: Immunogenicity and Safety of the Adjuvanted Recombinant Zoster Vaccine in Chronically Immunosuppressed Adults Following Renal Transplant: A Phase 3, Randomized Clinical Trial
Source: Clin Infect Dis. 2019 Mar 7;70(2):181–90. doi: 10.1093/cid/ciz177 (PMC6938982; doi:10.1093/cid/ciz177)
Supplement: ciz177_suppl_Supplementary_Materials [file ciz177_suppl_supplementary_materials.docx]

# Supplementary Materials

# Supplementary Text 1

# a) Inclusion criteria

- Patients able to comply with study procedures including follow up.
- ≥18 years of age, had reached the age of local legal consent and provided written informed consent.
- Received an ABO compatible allogeneic renal transplant.
- Received chronic immunosuppressive therapy for the prevention of allograft rejection for a minimum of 1 month (M) prior to the first vaccination.
- No allograft rejection over the previous 3M prior to the first vaccination.
- Stable renal function (<20% variability between last two creatinine measurements or calculated glomerular filtration rate [GFR] or in the opinion of the investigator, after investigator reviewed more than the last two creatinine measurements or calculated GFRs).
- Not less than 4M and not more than 18M after renal transplantation at the time of the first vaccination.
- Participants with multiple dialysis option.
- Female participant of non-childbearing potential (pre-menarche, current tubal ligation, hysterectomy, oophorectomy or post-menopause) and childbearing potential (if they had: practiced adequate contraception for 1M prior to vaccination, a negative pregnancy test on the day of the first vaccination and agreed to continue adequate contraception during the primary treatment period and for 2M after completion of the vaccination series).

# b) Exclusion criteria

- Any primary renal disease with a high incidence of recurrence in the transplant
- Evidence of recurrent primary renal disease within the current allograft.
- Previous allograft loss secondary to recurrent primary renal disease. Multiple renal transplants were allowed if the reason for a previous allograft’s loss is not recurrent primary renal disease.
- More than one organ transplanted (i.e. kidney-liver, double kidney or kidney-other organ(s) transplanted).
- History of events that, in the opinion of the investigator, may put the patient at increased risk for chronic allograft dysfunction (e.g. delayed graft function, peri-operative complications).
- Histologic reports of chronic allograft injury (e.g. transplant glomerulopathy, arteriopathy, C4d deposition).
- Evidence of significant proteinuria in the opinion of the investigator.
- Panel reactive antibody (PRA) or calculated PRA score that was unknown at the time of transplant.
- Any autoimmune or potential immune-mediated disease listed in **Supplementary Table 1**. Exceptions to autoimmune or potential immune-mediated diseases include: Immunoglobulin A nephropathy, glomerulonephritis rapidly progressive, membranous glomerulonephritis, idiopathic Type I membranoproliferative glomerulonephritis, diabetes mellitus (type 1 and 2) with diabetic nephropathy.
- Any confirmed or suspected human immunodeficiency virus infection, primary immunodeficiency disease, disseminated or untreated malignancy, or systemic infection.
- Use of anti-CD20 or other B-cell monoclonal antibody agents (e.g., rituximab) as induction, maintenance and/or therapeutic immunosuppressive therapy for the prevention of allograft rejection within 9M of first dose of study vaccine/placebo.
- Use of any investigational or non-registered product^‡^ (drug, vaccine or device) other than the study vaccine within 1M preceding the first dose of study vaccine/placebo, or planned use during the study period.
- Concurrent or planned participation in another clinical study, at any time during the study period, which had exposed or would have exposed the patient to an investigational or a non-registered product^‡^ (drug, vaccine or device).

^‡^Immunosuppressant(s), which are investigational or non-registered product(s) at the local/country level, were allowed to be used if they were specifically prescribed for the prevention of allograft rejection and were: (i) available locally through compassionate use programs, (ii) submitted for and pending local/country registration, (iii) approved and registered for use in other countries with well-documented Summary of Product Characteristics or Prescribing Information. The name of the active component(s) of these immunosuppressants had to be provided in the concomitant medication listing.

- Administration or planned administration of a live vaccine within 1M prior to the first dose of study vaccine and ending 1M after the last dose of study vaccine, or, administration or planned administration of a non-replicating vaccine (e.g. inactivated and subunit vaccines, including inactivated and subunit influenza vaccines and pneumococcal conjugate vaccines) within 8 days (D) prior to or within 14D after either dose of study vaccine.
- Planned administration during the study of a varicella or herpes zoster (HZ) vaccine (including an investigational or non-registered vaccine) other than the study vaccine.
- Previous vaccination against HZ or varicella within the 12M preceding the first dose of study vaccine/placebo.
- Occurrence of varicella or HZ per clinical history, within the 12M preceding the first dose of study vaccine/placebo.
- Failure to fully complete the 7D pre-vaccination diary card distributed at the pre-vaccination visit. Completion must cover the 7D immediately prior to the planned vaccination date. Full completion was defined as a minimum of 6D completed. Persons with less than 6D completed may have been offered a new vaccination date and the opportunity to fully complete the 7D pre-vaccination diary card over the last 7D prior to the new planned vaccination date.
- Evidence or high suspicion, in the opinion of the investigator, of noncompliance or nonadherence to use of induction and/or maintenance immunosuppressive therapies.
- History of any reaction or hypersensitivity likely to have been exacerbated by any component of the vaccine or study material and equipment.
- Any condition which, in the judgment of the investigator, would have made intramuscular injection unsafe.
- Any other condition that, in the opinion of the investigator, might have interfered with the evaluations required by the study.
- Acute disease and/or fever at the time of vaccination. Fever was defined as temperature ≥37.5°C / 99.5°F by oral route. The preferred route for recording temperature in this study was oral.
- Persons with a minor illness (such as mild diarrhea, mild upper respiratory infection) without fever may have been enrolled at the discretion of the investigator.
- Pregnant or lactating female.
- Female planning to become pregnant or planning to discontinue contraceptive precautions (if of childbearing potential) before Month 3 (i.e. 2M after last dose of study vaccine/placebo).

# Supplementary Text 2: Statistical methods

Vaccine response rate (VRR) in terms of anti-glycoprotein E (gE) humoral response was defined as the percentage of participants with post-vaccination anti-gE concentrations ≥4-fold the technical cut-off of assay quantification (for initially seronegative participants) or ≥4-fold the pre-vaccination concentration (for initially seropositive participants).

The geometric mean concentration (GMC) calculations were performed by taking the anti-log of the mean of the log concentration transformations. Antibody concentrations below the technical cut-off (TC) of assay quantification (97 mIU/mL) were given an arbitrary value equal to half the TC for the purpose of GMC calculation.

VRR in terms of CD4[2+] T-cell response was defined as the percentage of participants with post-vaccination CD4[2+] T-cell frequencies ≥2-fold the cut-off (320 positive cells per 10^6^ CD4+ T-cells counted) (for participants initially below the cut-off) or ≥2-fold the pre-vaccination CD4[2+] T-cell frequencies (for participants initially above the cut-off).

The general mean (GM) frequency calculations were performed by taking the anti-log of the mean of the log frequency transformations. For the inferential analysis, the frequency of CD4[2+] T-cells after *in vitro* stimulation with gE (induction condition) was calculated by adding an offset of 0.5 to the number of activated CD4[2+] T-cells divided by the total number of CD4+ T-cells involved. A similar calculation was used for the frequency of CD4[2+] T-cells after *in vitro* stimulation in medium only (background condition). For the descriptive analyses, the frequency of CD4[2+] T-cells upon *in vitro* stimulation with gE (induction condition) was calculated by dividing the number of activated CD4[2+] T-cells (numerator) over the total number of CD4 T-cells involved (denominator). The frequency of gE-specific CD4[2+] T-cells for each participant was calculated as the difference between the frequency of CD4[2+] T-cells, upon in vitro stimulation with gE (induction condition) minus the frequency of CD4[2+] T-cells upon in vitro stimulation in medium only (background condition). The differences ≤1 were imputed to 1 gE-specific CD4[2+] T cell per 10^6^ CD4+ T-cells.

All statistical analyses were performed using the Statistical Analysis Systems (SAS) version 9.3 on Windows Drug Development (SDD) Version 4.3.4.

*Sample size calculation*

Sample-size was calculated to provide 90% power for each confirmatory objective in the humoral endpoint. A 12.5-fold increase over placebo and a minimum of 3-fold increase in GM as lower limit was assumed for the analyses on all patients. A two-sided type 1 error of 5% was used. To obtain a power of at least 90% to see a minimum of 3-fold increase as lower limit in anti-gE humoral immune response over placebo, assuming a 12.5-fold increase between placebo and recombinant zoster vaccine (RZV) group, 100 evaluable patients per treatment group were needed. A sample size of 125 patients per group provided 94% power to detect a minimum of 60% VRR and minimum 3-fold increase in anti-gE humoral immune response over placebo assuming a 20% loss rate from drop-out and non-evaluable patients at Month 2.

Thirty participants per treatment arm were needed to provide 78% power to detect a significantly higher CD4[2+] T-cell frequency in RZV than in placebo recipients. Assuming a non-evaluability rate of 20% from drop-out and non-evaluable patients at Month 2, 38 participants from each treatment arm had to be assigned to the cell-mediated immunogenicity sub-cohort.

# Supplementary Table 1. Pre-specified potential immune-mediated diseases included in the protocol

| **Musculoskeletal disorders** | **Skin disorders** | **Neuroinflammatory disorders** |
| --- | --- | --- |
| - - - - Systemic lupus erythematosus and associated conditions       - Systemic scleroderma (Systemic sclerosis), including diffuse systemic form and CREST syndrome       - Idiopathic inflammatory myopathies, including dermatomyositis, polymyositis       - Antisynthetase syndrome       - Rheumatoid arthritis and associated conditions including juvenile chronic arthritis and Still’s disease)       - Polymyalgia rheumatic       - Spondyloarthritis (including ankylosing spondylitis, reactive arthritis [Reiter's Syndrome] and undifferentiated spondyloarthritis)       - Psoriatic arthropathy       - Relapsing polychondritis       - Mixed connective tissue disorder | - Psoriasis - Vitiligo - Erythema nodosum - Autoimmune bullous skin diseases (including pemphigus, pemphigoid and dermatitis herpetiformis) - Alopecia areata - Lichen planus - Sweet’s syndrome - Localized Scleroderma (Morphoea) | - Cranial nerve disorders, including paralyses/paresis (e.g. Bell’s palsy), and neuritis (e.g. optic neuritis) - Multiple sclerosis (including variants) - Transverse myelitis - Guillain-Barré syndrome (including Miller Fisher syndrome and other variants) - Acute disseminated encephalomyelitis (including site specific variants: e.g. noninfectious encephalitis, encephalomyelitis, myelitis, myeloradiculoneuritis) - Myasthenia gravis (including Lambert-Eaton myasthenic syndrome) - Immune-mediated peripheral neuropathies and plexopathies, (including chronic inflammatory demyelinating polyneuropathy, multifocal motor neuropathy and polyneuropathies associated with monoclonal gammapathy). - Narcolepsy |
| **Gastrointestinal disorders** | **Liver disorders** | **Endocrine disorders** |
| - Inflammatory Bowel disease, including Crohn’s disease, ulcerative colitis, microscopic colitis, ulcerative proctitis - Celiac disease - Autoimmune pancreatitis | - Autoimmune hepatitis - Primary biliary cirrhosis - Primary sclerosing cholangitis - Autoimmune cholangitis | - Autoimmune thyroiditis (including Hashimoto thyroiditis) - Grave's or Basedow’s disease - Diabetes mellitus type I - Addison’s disease - Polyglandular autoimmune syndrome - Autoimmune hypophysitis |
| **Vasculitis** | **Blood disorders** | **Others** |
| - Large vessels vasculitis including: giant cell arteritis such as Takayasu's arteritis and temporal arteritis. - Medium sized and/or small vessels vasculitis including: polyarteritis nodosa, Kawasaki's disease, microscopic polyangiitis, Wegener's granulomatosis, Churg–Strauss syndrome (allergic granulomatous angiitis), Buerger’s disease (thromboangiitis obliterans), necrotizing vasculitis and antineutrophil cytoplasmic antibody positive vasculitis (type unspecified), Henoch-Schonlein purpura, Behcet's syndrome, leukocytoclastic vasculitis. | - Autoimmune hemolytic anemia - Autoimmune thrombocytopenia - Antiphospholipid syndrome - Pernicious anemia - Autoimmune aplastic anemia - Autoimmune neutropenia - Autoimmune pancytopenia | - Autoimmune glomerulonephritis (including Immunoglobulin A nephropathy, glomerulonephritis rapidly progressive, membranous glomerulonephritis, membranoproliferative glomerulonephritis, and mesangioproliferative glomerulonephritis) - Ocular autoimmune diseases (including autoimmune uveitis and autoimmune retinopathy) - Autoimmune myocarditis/cardiomyopathy - Sarcoidosis - Stevens-Johnson syndrome - Sjögren’s syndrome - Idiopathic pulmonary fibrosis - Goodpasture syndrome - Raynaud’s phenomenon |

# **Supplementary Table 2.** Humoral immune responses to RZV by type of immunosuppressive therapy (ATP cohort for humoral immunogenicity)

|  |  | **Anti-gE antibody GMC** | | | | **Humoral VRR** | | | |
| --- | --- | --- | --- | --- | --- | --- | --- | --- | --- |
|  |  | **RZV** | | **Placebo** | | **RZV** | | **Placebo** | |
| **Immunosuppressive therapy** | **Time point** | **N** | **Value (95%CI)** | **N** | **Value (95%CI)** | **N** | **% (95%CI)** | **N** | **% (95%CI)** |
| **CIS + CS+ MC** | **M0** | 92 | 1394.7 (1119.4–1737.9) | 91 | 1435.3 (1116.6–1844.9) | - | - | - | - |
|  | **M1** | 92 | 8764.1 (6173.9–12441.1) | 91 | 1439.9 (1147.7–1806.6) | 92 | 58.7 (47.9–68.9) | 91 | 2.2 (0.3–7.7) |
|  | **M2** | 92 | 18164.8 (13549.0–24353.2) | 91 | 1442.2 (1142.5–1820.5) | 92 | 77.2 (67.2–85.3) | 91 | 4.4 (1.2–10.9) |
|  | **M7** | 84 | 12239.3 (9265.3–16167.9) | 86 | 1525.5 (1201.9–1936.3) | 84 | 72.6 (61.8–81.8) | 86 | 4.7 (1.3–11.5) |
|  | **M13** | 83 | 7739.9 (5836.8–10263.5) | 86 | 1556.7 (1215.2–1994.1) | 83 | 62.7 (51.3–73.0) | 85 | 8.2 (3.4–16.2) |
| **CIS + MC** | **M0** | 21 | 1144.5 (732.9–1787.2) | 20 | 1708.4 (935.0–3121.6) | - | - | - | - |
|  | **M1** | 21 | 10594.3 (5885.6–19070.1) | 20 | 1750.5 (1013.1–3024.8) | 21 | 76.2 (52.8–91.8) | 20 | 5.0 (0.1–24.9) |
|  | **M2** | 21 | 23078.4 (14901.9–35741.3) | 20 | 1677.6 (967.9–2907.4) | 21 | 90.5 (69.6–98.8) | 20 | 5.0 (0.1–24.9) |
|  | **M7** | 19 | 17470.6 (11083.4–27538.6) | 21 | 1553.1 (918.4–2626.4) | 19 | 89.5 (66.9–98.7) | 20 | 5.0 (0.1–24.9) |
|  | **M13** | 20 | 10847.3 (7053.7–16681.2) | 18 | 1706.8 (970.6–3001.2) | 20 | 75.0 (50.9–91.3) | 17 | 0.0 (0.0–19.5) |
| **CIS + CS** | **M0** | 6 | 2040.1 (603.3–6898.9) | 8 | 1715.5 (708.5–4153.8) | - | - | - | - |
|  | **M1** | 6 | 14961.1 (2574.1–86956.3) | 8 | 1653.6 (678.7–4028.6) | 6 | 83.3 (35.9–99.6) | 8 | 0.0 (0.0–36.9) |
|  | **M2** | 6 | 20507.0 (3907.4–107625.7) | 8 | 1596.0 (641.3–3971.9) | 6 | 83.3 (35.9–99.6) | 8 | 0.0 (0.0–36.9) |
|  | **M7** | 6 | 16558.2 (2392.6–114590.2) | 8 | 1571.0 (628.0–3929.9) | 6 | 66.7 (22.3–95.7) | 8 | 0.0 (0.0–36.9) |
|  | **M13** | 6 | 15821.5 (3131.5–79935.6) | 7 | 1444.6 (533.3–3913.5) | 6 | 83.3 (35.9–99.6) | 7 | 0.0 (0.0–41.0) |

gE, glycoprotein E; GMC, geometric mean concentration; VRR, vaccine response rate; RZV, participants receiving the recombinant adjuvanted zoster vaccine; Placebo, participants receiving placebo; ATP, according-to-protocol; CIS, calcineurin inhibitor or sirolimus; CS, corticosteroids; MC, mycophenolate compound; N, number of participants with available results; 95%CI, 95% confidence interval; M, study month; M0, pre-vaccination; M1, 1 month post-dose 1; M2, 1 month post-dose 2; M7, 6 months post-dose 2; M13, 12 months post-dose 2.

Note: Some combinations of maintenance immunosuppressive therapy used at Visit 1 are not displayed due to limited group size (N ≤2).

#

# Supplementary Table 3. Geometric mean concentrations of anti-gE antibody and vaccine response rates for anti-gE antibody ELISA concentrations by age strata (post-hoc analyses, ATP cohort for humoral immunogenicity)

|  |  | **Anti-gE antibody GMC** | | | | | **Humoral VRR** | | |
| --- | --- | --- | --- | --- | --- | --- | --- | --- | --- |
|  |  | **RZV** | | | **Placebo** | | **RZV** | | **Placebo** |
| **YOA** | **Time point** | **N*** | **Value (95%CI)** | **N*** | **Value (95%CI)** | **N*** | **% (95%CI)** | **N*** | **% (95%CI)** |
| **18 -29** | **M0** | 6 | 703.3 (133.0–3718.5) | 6 | 1265.1 (220.6–7256.7) | - | - | - | - |
|  | **M1** | 6 | 25623.4 (5445.0–120579.4) | 6 | 1703.1 (335.3–8650.4) | 6 | 100 (54.1–100) | 6 | 16.7 (0.4–64.1) |
|  | **M2** | 6 | 47416.8 (14421.3–155905.7) | 6 | 1524.0 (298.2–7787.7) | 6 | 100 (54.1–100) | 6 | 16.7 (0.4–64.1) |
|  | **M7** | 6 | 24973.2 (6427.9–97023.7) | 6 | 1305.4 (294.0–5796.7) | 6 | 100 (54.1–100) | 6 | 0.0 (0.0–45.9) |
|  | **M13** | 6 | 16719.0 (3877.9–72082.2) | 6 | 1362.5 (334.9–5542.9) | 6 | 83.3 (35.9–99.6) | 6 | 16.7 (0.4–64.1) |
| **30-49** | **M0** | 40 | 1771.4 (1193.6–2628.8) | 35 | 2166.7 (1556.8–3015.4) | - | - | - | - |
|  | **M1** | 40 | 16927.8 (9102.7–31479.7) | 35 | 2061.4 (1511.7–2811.1) | 40 | 70.0 (53.5–83.4) | 35 | 0.0 (0.0–10.0) |
|  | **M2** | 40 | 28009.7 (17296.7–45357.9) | 35 | 1963.6 (1446.3–2665.9) | 40 | 82.5 (67.2–92.7) | 35 | 0.0 (0.0–10.0) |
|  | **M7** | 35 | 18817.0 (11340.0–31224.0) | 33 | 2199.3 (1547.1–3126.5) | 35 | 77.1 (59.9–89.6) | 33 | 3.0 (0.1–15.8) |
|  | **M13** | 35 | 11393.8 (6990.9–18569.6) | 34 | 2418.4 (1705.9–3428.5) | 35 | 68.6 (50.7–83.1) | 33 | 6.1 (0.7–20.2) |

gE, glycoprotein E; YOA, years of age; GMC, geometric mean concentration; VRR, vaccine response rate; RZV, participants receiving the recombinant adjuvanted herpes zoster vaccine; Placebo, participants receiving placebo; ATP, according-to-protocol; M, study month; M0, pre-vaccination; M1, 1 month post-dose 1; M2, 1 month post-dose 2; M7, 6 months post-dose 2; M13, 12 months post-dose 2; N, number of participants with pre- and post-vaccination results available; %, percentage of responders; 95% CI, 95% confidence interval; *, These results should be interpreted with caution due to the very small sample size.

Vaccine response rate in terms of anti-gE humoral response was defined as the percentage of participants with post-vaccination anti-gE concentrations (i) ≥4-fold the technical cut-off of assay quantification (for initially seronegative participants) or (ii) ≥4-fold the pre-vaccination concentration (for initially seropositive participants).

# Supplementary Table 4. Descriptive statistics of the frequency of gE-specific CD4[2+] T-cells and vaccine response rates for gE-specific CD4[2+] T-cell frequencies by age strata (post-hoc analyses, ATP cohort for CMI)

|  |  | **CD4[2+] T-cells frequencies** | | | | | | | | | | | | **CMI VRR** | | | |
| --- | --- | --- | --- | --- | --- | --- | --- | --- | --- | --- | --- | --- | --- | --- | --- | --- | --- |
|  |  | **RZV** | | | | | | **Placebo** | | | | | | **RZV** | | **Placebo** | |
| **YOA** | **Time point** | **N*** | **Min** | **Q1** | **Median** | **Q3** | **Max** | **N*** | **Min** | **Q1** | **Median** | **Q3** | **Max** | **N*** | **% (95%CI)** | **N*** | **% (95%CI)** |
| **18 -29** | **M0** | 2 | 41.0 | 41.0 | 58.5 | 76.0 | 76.0 | 1 | 1.0 | 1.0 | 1.0 | 1.0 | 1.0 | - | - | - | - |
|  | **M2** | 2 | 2311.1 | 2311.1 | 2635.2 | 2959.3 | 2959.3 | 1 | 268.4 | 268.4 | 268.4 | 268.4 | 268.4 | 2 | 100 (15.8–100) | 1 | 0.0 (0.0–97.5) |
|  | **M13** | 2 | 973.5 | 973.5 | 3729.2 | 6484.9 | 6484.9 | 1 | 193.2 | 193.2 | 193.2 | 193.2 | 193.2 | 2 | 100 (15.8–100) | 1 | 0.0 (0.0–97.5) |
| **30-49** | **M0** | 10 | 1.0 | 11.7 | 70.4 | 210.2 | 418.5 | 11 | 1.0 | 66.5 | 301.2 | 487.1 | 684.2 | - | - | - | - |
|  | **M2** | 10 | 499.5 | 824.7 | 2932.3 | 3827.6 | 4798.1 | 9 | 1.0 | 43.1 | 135.0 | 189.7 | 244.1 | 9 | 77.8 (40.0–97.2) | 9 | 0.0 (0.0–33.6) |
|  | **M13** | 11 | 1.0 | 498.7 | 1247.2 | 1765.2 | 9230.7 | 10 | 1.0 | 33.4 | 152.2 | 367.3 | 574.1 | 10 | 70.0 (34.8–93.3) | 10 | 0.0 (0.0–30.8) |

YOA, years of age; VRR, Vaccine response rate; RZV, participants receiving the recombinant adjuvanted herpes zoster vaccine; Placebo, participants receiving placebo; ATP, according-to-protocol; CMI, cell-mediated immunogenicity; M, study month; M0, pre-vaccination; M2, 1 month post-dose 2; M13, 12 months post-dose 2; N, number of participants with available results; SD, Standard Deviation; Q1, Q3, First and third quartiles; Min/Max, Minimum/Maximum; *, These results should be interpreted with caution due to the very small sample size.

Vaccine response rate in terms of CD4[2+] T-cell response was defined as the percentage of participants with post-vaccination CD4[2+] T-cell frequencies (i) ≥2-fold the cut-off (320 positive cells per 106 CD4+ T-cells counted) (for participants initially below the cut-off) or (ii) ≥2-fold the pre-vaccination CD4[2+] T-cell frequencies (for participants initially above the cut-off)

# Supplementary Figure 1. Reactogenicity (TVC)
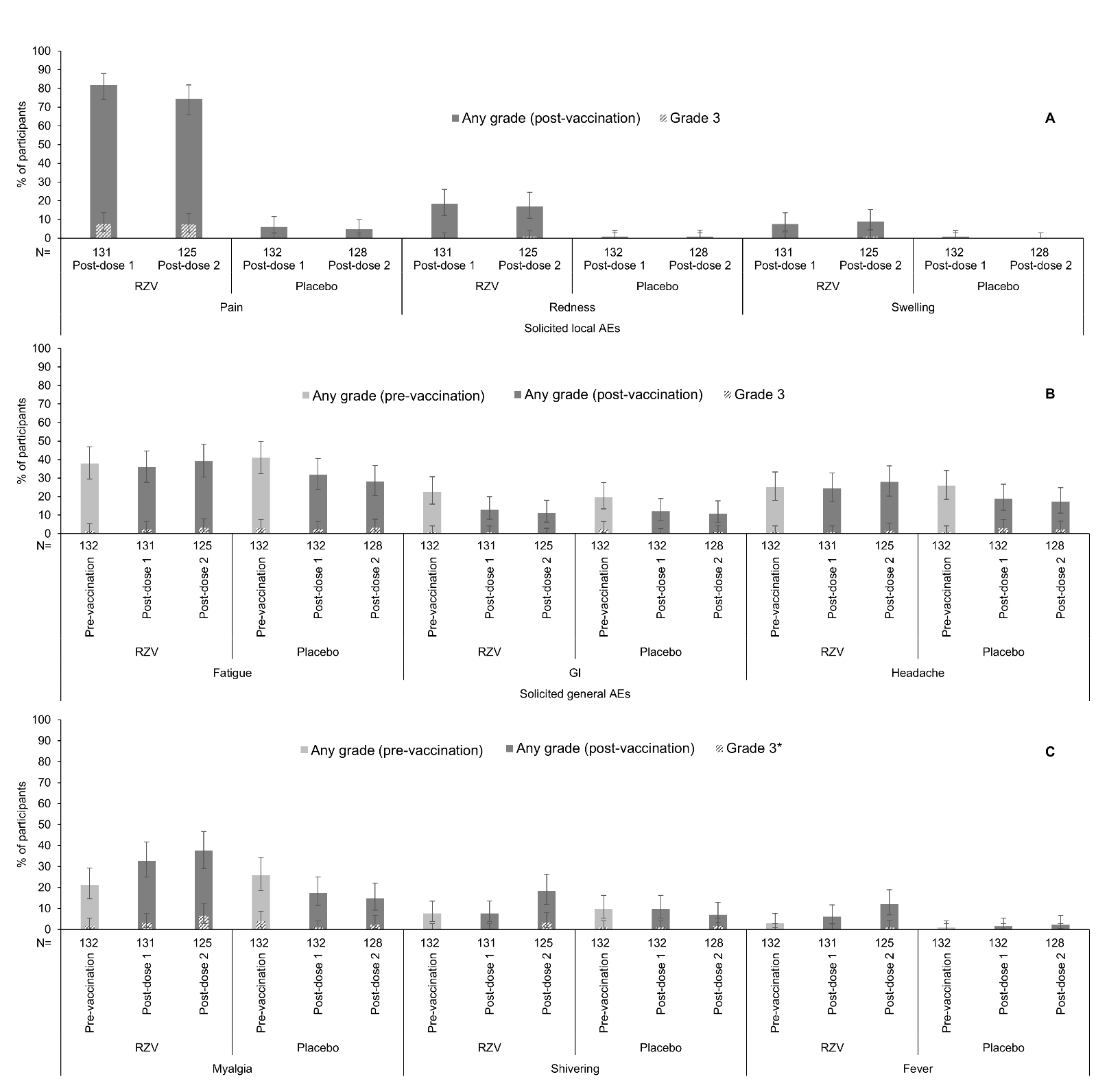


**A.** Solicited local AEs reported within 7 days post-vaccination

**B** and **C.** Solicited general AEs reported within 7 days before the first dose (background condition) and within 7 days post-vaccination

RZV, participants receiving the recombinant adjuvanted herpes zoster vaccine; Placebo, participants receiving placebo; TVC, total vaccinated cohort, N, number of participants with at least one documented vaccine administration; GI, gastrointestinal symptoms (nausea, vomiting, diarrhea and/or abdominal pain).

Fever was defined as body temperature ≥37.5 °C. *Fever was not graded in this study. For fever, body temperatures >39 °C are presented in this category.
